# Patient Aggression and the Wellbeing of Nurses: A Cross-Sectional Survey Study in Psychiatric and Non-Psychiatric Settings

**DOI:** 10.3390/ijerph14101245

**Published:** 2017-10-18

**Authors:** Virve Pekurinen, Laura Willman, Marianna Virtanen, Mika Kivimäki, Jussi Vahtera, Maritta Välimäki

**Affiliations:** 1 Department of Nursing Science, University of Turku, 20520 Turku, Finland; vimapek@utu.fi (V.P.); laura.k.willman@utu.fi (L.W.); 2 Finnish Institute of Occupational Health, 00250 Helsinki, Finland; marianna.virtanen@ttl.fi (M.V.); mika.kivimaki@helsinki.fi (M.K.); 3 Department of Epidemiology and Public Health, University College London, London WC1E 7HB, UK; 4 Clinicum, Faculty of Medicine, University of Helsinki, 00290 Helsinki, Finland; 5 Department of Public Health, University of Turku, 20520 Turku, Finland; jussi.vahtera@utu.fi; 6 Turku University Hospital, 20521 Turku, Finland; 7 School of Nursing, Hong Kong Polytechnic University, Hong Kong, China

**Keywords:** psychiatric nurses, non-psychiatric nurses, occupational health, psychological distress, self-rated health, sleep disturbance, work ability, patient aggression

## Abstract

Wellbeing of nurses is associated with patient aggression. Little is known about the differences in these associations between nurses working in different specialties. We aimed to estimate and compare the prevalence of patient aggression and the associations between patient aggression and the wellbeing of nurses in psychiatric and non-psychiatric specialties (medical and surgical, and emergency medicine). A sample of 5288 nurses (923 psychiatric nurses, 4070 medical and surgical nurses, 295 emergency nurses) participated in the study. Subjective measures were used to assess both the occurrence of patient aggression and the wellbeing of nurses (self-rated health, sleep disturbances, psychological distress and perceived work ability). Binary logistic regression with interaction terms was used to compare the associations between patient aggression and the wellbeing of nurses. Psychiatric nurses reported all types of patient aggression more frequently than medical and surgical nurses, whereas nurses working in emergency settings reported physical violence and verbal aggression more frequently than psychiatric nurses. Psychiatric nurses reported poor self-rated health and reduced work ability more frequently than both of the non-psychiatric nursing groups, whereas medical and surgical nurses reported psychological distress and sleep disturbances more often. Psychiatric nurses who had experienced at least one type of patient aggression or mental abuse in the previous year, were less likely to suffer from psychological distress and sleep disturbances compared to medical and surgical nurses. Psychiatric nurses who had experienced physical assaults and armed threats were less likely to suffer from sleep disturbances compared to nurses working in emergency settings. Compared to medical and surgical nurses, psychiatric nurses face patient aggression more often, but certain types of aggression are more common in emergency settings. Psychiatric nurses have worse subjective health and work ability than both of the non-psychiatric nursing groups, while their psychiatric wellbeing is better and they have less sleep problems compared to medical and surgical nurses. Psychiatric nurses maintain better psychiatric wellbeing and experience fewer sleep problems than non-psychiatric nurses after events of exposure to patient aggression. This suggest that more attention should be given to non-psychiatric settings for maintaining the wellbeing of nurses after exposure to patient aggression.

## 1. Introduction

Patient aggression toward health professionals is a serious global concern [1,2]. Health professionals taking care of persons with mental disturbances are often exposed to patient aggression [2]. Aggression can be defined as a range of behaviors or actions that has the potential to harm, hurt or injure another person, either physically or verbally, regardless of whether or not harm is actually sustained or the intention is clear [3]. Patient aggression in these settings is associated with healthcare workers’ wellbeing [4,5,6]. Being the target of patient aggression has been found to be associated with anxiety, fear, guilt, sleep disturbances [7], burnout [8,9], poor self-rated health [10] or dissatisfaction toward work [4]. Furthermore, longitudinal studies have shown that the relationship between workplace aggression and the wellbeing of employees seems bidirectional; those who experience aggression are more likely to report occupational stress, and those who report occupational stress are at a higher risk of workplace aggression [11,12].

Patient aggression toward nurses has been documented in several empirical studies (e.g., [13,14,15,16]). Staff members working in mental health settings are at a higher risk of being assaulted by patients [2,17]. For example, a systematic review [2] showed that the rate of physical violence varied considerably across settings, the highest being in psychiatry (55%). The risk for aggression may be greater among inpatients, persons with substance abuse disorder [18] and those who have severe mental disorders [19,20]. A study conducted on a self-selected sample of psychiatric wards in the Veneto Region of Italy [21] found that nearly two-thirds (66.4%, N = 2017) of the staff who worked in psychiatry had a high level of job distress, and nearly one-fifth (19.6%, N = 281) suffered from severe burnout. Working in psychiatry also includes greater odds for diagnosed depression, antidepressant medication use and sick leave due to depression and mental disorders [22]. On the other hand, staff working in emergency care units are at an elevated risk of experiencing physical aggression, although the risk is lower than for staff working in psychiatric settings [2,17]. The risk of experiencing physical aggression is significantly lower in medical and surgical specialties [17].

To prevent a serious shortage of nurses in the coming years [23] and nurses leaving the field because of increased stress as a result of patient aggression [4,8], more knowledge about the association between patient aggression and nurses’ wellbeing is needed. As the data presented in this article is part of a larger data set (see, e.g., [24,25]), we report the results of the survey of a representative sample for nurses working in psychiatric and non-psychiatric settings (medical and surgical, and emergency specialties). We aim to estimate and compare the prevalence of patient aggression and the associations between patient aggression and the wellbeing of nurses in psychiatric settings and the two specified non-psychiatric nursing environments. We hypothesize that (a) more nurses working in psychiatric settings experience patient aggression than nurses in non-psychiatric settings; (b) nurses working in psychiatric settings have poorer self-rated health, more sleep disturbances and psychological distress, and reduced work ability compared to nurses in non-psychiatric settings; and (c) nurses who experience patient aggression while working in psychiatric settings are more likely to experience poor self-rated health, sleep disturbances, psychological distress and reduced work ability compared to their counterparts in non-psychiatric settings.

## 2. Materials and Methods

### 2.1. Design and Data Collection

The cross-sectional study data is based on a subset of a Finnish Public Sector study (FPS [24]), and the survey was collected in the form of questionnaires in 2012. In Finland, specialized health services are mostly public, tax-funded, and organized by hospital districts responsible for specialized care in their area [26]. In Finland, universities of applied sciences offer bachelor-level education for registered nurses (RNs) and other nursing-based professions, while vocational schools educate practical nurses [27]. Head nurses are educated either as specialized nurses or they may possess a master’s degree in Health Sciences, depending on the organization. The cultural background of nurses is quite homogenous; in 2013, less than 4% of registered nurses were immigrants, while the corresponding number regarding practical nurses was slightly more than 5% [28].

Since the year 2000, employers’ records have been used to identify employees eligible for nested survey cohorts in the FPS study. Employees have subsequently been sent questionnaires by e-mail or mail every four years. This survey was carried out in 2012, and it included employees of 21 public hospitals in five hospital districts and one regional hospital. Employers’ records were used to identify eligible employees. Potential participants included all working nurses (registered nurses or practical/mental health nurses) from a variety of medical specialties in the participating hospitals at the time of the data collection. Answering the questionnaire was considered to signify informed consent (Medical Research Act 9.4.1999/488). A total of 7523 nurses (1033 psychiatric nurses and 6490 non-psychiatric nurses) received the questionnaire and an invitation to participate in the study, and 5228 returned the completed questionnaire (response rate 70%). The Ethics Committee of the Hospital District of Helsinki and Uusimaa assessed the study (60/13/03/00/2011) and the hospital organizations approved the study.

### 2.2. Measures

Patient aggression was assessed retrospectively [29]. Respondents were asked to state if they had encountered any of the four types of patient aggression at work during the previous 12 months (0 = no, 1 = yes): (1) mental abuse (such as verbal threats), (2) physical violence (such as hitting or kicking), (3) assaults on ward property (such as throwing or breaking objects), or (4) armed threats with a firearm, edged weapon, or striking weapon [29]. In addition, the overall exposure to patient aggression was specified if the respondent had faced any of the four types of aggression (“1”). The measure has been used previously to assess the occurrence of aggression at work, not only regarding healthcare workers (e.g., [29]), but also with employees working in other sectors, e.g., basic education in Finland [30,31]. The internal consistency of the measure has been found to be acceptable in the field of psychiatric nursing (KR20 0.77 [25]), and it remained acceptable in this sample (KR20 = 0.77).

Self-rated health was measured with a widely-used self-rated scale. A single item question assessed perceived health status using a 5-point scale (1 = good, 2 = rather good, 3 = average, 4 = rather poor and 5 = poor). As in earlier studies (e.g., [32,33]), nurses’ health status was recategorized as a dichotomized rating (“good” or “rather good” = “good”; “average,” “rather poor” or “poor” = “poor”). The measure has been shown to be sensitive to changes in health status [34], to predict future mortality [32] and to reflect mental health [35].

Psychological distress was measured with the 12-item version of the General Health Questionnaire (GHQ [36,37]). The GHQ-12 is a self-administered screening instrument for common mental disorders and psychiatric wellbeing reflecting the level of psychological distress. The instrument focuses on anxiety, depression, social interaction, and self-confidence. Respondents rate how often they have experienced the symptoms of distress described in the items in the past few weeks on a four-point scale (0 = not at all, 1 = same as usual, 2 = slightly more than usual, 3 = much more than usual); the higher the score, the greater the psychological distress. We used a bimodal scoring method where “less than usual” and “no more than usual” were recalculated as “0,” and “slightly more than usual” and “much more than usual” were recalculated as “1” (possible sum score 0–12). As recommended in the validation study of the GHQ-12 regarding the Finnish population, a threshold of ¾ (0–3 = no psychological distress, 4–12 = psychological distress) was used in our study to identify nurses with psychological distress [38]. A threshold of ¾ has demonstrated excellent sensitivity (81.7) and specificity (85.4), and it has been recommended for use in public mental health surveys [39]. 

Sleep disturbances among nurses during the four weeks prior to the measurement were assessed with the Jenkins Sleep Scale [40], a widely used brief self-report instrument [41]. Corresponding to the nighttime insomnia symptoms specified by the Diagnostic and Statistical Manual of Mental Disorders (Fourth Edition, DSM-IV), respondents were asked to rate four items: (1) how often they had trouble falling asleep; (2) if they woke up several times per night, (3) if they had trouble staying asleep including waking up too early; and (4) if they felt tired after a normal night’s sleep. A five-point ordinal scale was employed (1 = never, 2 = 1–3 nights a month, 3 = approximately 1 night a week, 4 = 2–4 nights a week, 5 = 5–6 nights a week, 6 = every night). Those who scored any of the four sleep problems with a 4 or higher, were coded as having sleep disturbances (e.g., [42]).

The assessment of perceived work ability was based on responses to a single-item regarding nurses’ perceptions of their current work ability compared with the lifetime best. The item was derived from the Work Ability Index (WAI) developed by FIOH [43]. The respondents were asked to rate their work ability on a scale ranging from 0 (“completely unable to work”) to 10 (“work ability at its best”). This single item has been found to be reliable and comparable with the validity of the original Work Ability Index [44,45]. As has previously been done (e.g., [46]), perceived work ability was dichotomized into good (8–10 points) and reduced (0–7 points).

In addition, demographic information about the nurses was collected. We collected information on occupation (practical nurse, registered nurse/specialized nurse, head nurse), type of employment relationship (permanent, temporary), hospital district, and unit medical specialty from the employers’ registers. Participating hospitals encompassed 15 specialties, which were categorized first as psychiatric or non-psychiatric specialties. Further, we divided non-psychiatric specialties into two groups: medical and surgical, and emergency medicine. Medical and surgical specialties consisted of the following specialties: internal medicine, pediatrics, surgery, intensive care, pulmonary diseases, ophthalmology, otology, neurology, dermatology and venereology, oncology, physiatry, obstetrics and gynecology. Emergency medicine consisted of nurses working in emergency and ambulatory services. From the survey, the following demographic information was collected: gender, number of years working at the current hospital and current position, nature of work (full-time, part-time) and form of regular working hours (regular daytime work, two shifts, three shifts, night shift only, other irregular work).

### 2.3. Statistical Analysis

The description of the data was carried out using frequency distributions and variable statistics. Fisher’s exact test was used to analyze the comparison of the exposure to patient aggression between the psychiatric and non-psychiatric nurses (nurses working in medical and surgical specialties, and emergency medicine). Pearson correlation was used to examine how the wellbeing scores are related, and comparisons of wellbeing were analyzed using cross-tabulations and a Chi-squared test (*x*2). Effect sizes were calculated as Cramer’s V. Binary logistic regression models were used to compare the differences in the associations of different types of patient aggression and the various indicators of nurses’ wellbeing in psychiatric and the two non-psychiatric specialties [47]. For each of the models, there was a binary response (yes, no) for each wellbeing outcome variable (self-rated health, psychological distress, sleep disturbances and work ability). For predictive variables, we included an interaction term between medical specialty (psychiatric and medical and surgical specialties, or emergency medicine) and experiences of aggression (yes), to allow the comparison between the wellbeing outcomes of patient aggression between the specialty groups. In each of the models, the psychiatric nurses who had experienced different types of patient aggression were compared to one of the two groups of non-psychiatric nurses who had experienced patient aggression. However, there were too few observations to study the interactions regarding psychiatric nurses and emergency nurses who had experienced armed threats. Therefore, as done previously [29], we combined two aggression types: armed threats and physical assaults. We used this indicator in our analysis to compare the differences in the associations of physical assaults and armed threats and the various indicators of nurses’ wellbeing in psychiatric and emergency specialties. Finally, we controlled the models for gender and occupation, due to differences in these demographics in the nursing groups.

The results are presented as odd ratios (ORs) and their 95% confidence intervals (Cis). In addition, Wald statistics with degrees of freedom (df) and *p*-values are presented. In all tests, *p*-values of <0.05 were considered to indicate a statistically significant difference. Analyses were undertaken using SPSS version 22.0 (SPSS IBM, New York, NY, USA).

## 3. Results

### 3.1. Description of the Demographic Information

Out of 5288 nurses, 923 nurses worked in psychiatric settings. In the non-psychiatric settings, 4070 worked in medical and surgical settings and 295 worked in emergency settings. In all three groups, the majority of the nurses worked full-time and had a permanent contract. More detailed demographic information of the nurses in both groups is presented in Table 1.

### 3.2. Patient Aggression in Psychiatric and Non-Psychiatric Settings

In our data (N = 5228), 41% had experienced at least one type of aggression by patients within the previous 12 months. About one-third (37%) had experienced mental abuse, 25% physical violence and 21% assaults on ward property. The rarest type of patient aggression was that of armed threats (2%). Table 2 shows the comparisons of nurses’ exposure to different types of patient aggression in psychiatric and non-psychiatric settings.

We first hypothesized that more nurses working in psychiatric settings experience patient aggression than nurses in non-psychiatric settings. Our study hypothesis was partially supported: nurses working in psychiatric settings experienced all of the individual types of patient aggression (assaults on ward property, mental abuse, physical assaults and armed threats, *p* < 0.001) more often than nurses in medical and surgical settings. We also found that nurses working in psychiatric settings have experienced at least one type of patient aggression (overall) within the previous 12 months more often than nurses in medical and surgical settings (psychiatric nurses 65% vs. medical and surgical nurses 36%, *p* < 0.001). However, when we looked at nurses’ experiences of aggression in psychiatric settings compared to those of nurses in emergency settings, we found that nurses working in emergency settings had experienced at least one type of patient aggression (overall) within the previous 12 months more often than psychiatric nurses. Also, physical violence and mental abuse were found to happen more often in emergency settings.

### 3.3. The Wellbeing of Nurses

Out of all nurses (N = 5288), 17% rated their health as poor. Similarly, about one-fifth (21%) suffered from psychological distress and reduced work ability (21%), while a little less than half (49%) suffered from sleep disturbances. Table 3 presents the moderate correlations between the wellbeing scores.

Our second hypothesis was that nurses working in psychiatric settings have poorer self-rated health, more sleep disturbances, psychological distress and reduced work ability compared to nurses in non-psychiatric settings. Table 4 presents the results of cross-tabulations, Chi-squared test (x^2^) with effect sizes, and mean values of the wellbeing scores. We found statistically significant differences in the wellbeing scores among nurses working in psychiatric and those working in non-psychiatric settings. First, a higher number of nurses working in psychiatric settings had both poor self-rated health and reduced work ability compared to nurses working in medical and surgical settings (20% vs. 16%, *p* = 0.012 and 25% vs. 20%, *p* = 0.003). On the other hand, psychological distress and disturbed sleep were more common among nurses working in medical and surgical settings (22% vs. 19%, *p* = 0.019, and 51% vs. 43%, *p* < 0.001, respectively). Second, a higher number of nurses working in psychiatric settings had both poor self-rated health and reduced work ability compared to nurses working emergency settings (20% vs. 12%, *p* = 0.002 and 25% vs. 13%, *p* < 0.001). However, the differences between the mean values of these scores in psychiatric and the both non-psychiatric settings were small, as were the effect sizes (Table 4). Thus, our second hypothesis was only partially supported by our study results.

### 3.4. Comparison of Associations between Patient Aggression and the Wellbeing of Nurses Working in Psychiatric and Non-Psychiatric Settings

Our third hypothesis was that nurses who work in psychiatric settings and experience patient aggression are more likely to have poor self-rated health, sleep disturbances, psychological distress and reduced work ability compared to their counterparts in non-psychiatric settings.

The analysis showed first that nurses in psychiatric settings who had experienced at least one type of patient aggression in the previous 12 months were less likely to suffer from psychological distress and sleep disturbances compared to nurses working in medical and surgical settings (OR 0.55, test of interaction *p* = 0.003 and OR 0.65, test of interaction *p* = 0.007, respectively). Similarly, nurses working in psychiatric settings who had experienced mental abuse were again less likely to suffer from psychological distress and sleep disturbances compared to nurses working in medical and surgical settings (OR 0.39, test of interaction *p* < 0.001). Table 5 presents the results of these logistic regression models with interaction terms.

Regarding comparisons between nurses in psychiatric and emergency settings, nurses working in psychiatric settings who had been subjected to physical assaults and armed threats were less likely to experience sleep disturbances compared to nurses working in emergency settings (OR 0.57, test of interaction *p* = 0.044). Table 6 presents the results of these logistic regression models with interaction terms.

All of the interactions remained significant after controlling for gender and occupational level. We found no statistically significant interactions between psychiatric and the two non-psychiatric settings regarding work ability or any of the different types of patient aggression. Thus, our third hypothesis was not supported by our study results.

## 4. Discussion

In our cross-sectional survey among nurses in different settings, we found that more nurses in psychiatric settings experienced patient aggression compared to nurses who worked in medical and surgical settings. The finding is in line with previous studies [2,17]. However, we also found that physical aggression and mental abuse were more common in emergency settings, compared to psychiatric settings. The finding regarding physical aggression is not totally in line with previous studies, although earlier research has reported emergency settings as having a high risk for experiencing physical aggression [2,17]. On the other hand, some studies have found a higher occurrence of non-physical aggression in emergency settings, compared to psychiatric settings [2]. Nevertheless, the finding regarding the high occurrence of patient aggression in psychiatric settings is worrying because working in psychiatry includes higher odds for diagnosed depression, antidepressant medication use and sick leave due to depression and mental disorders [22].

Contrary to our preliminary assumption, we found that nurses working in medical and surgical settings suffer from psychological distress and sleep disturbances more often than nurses in psychiatric settings, whereas we did not detect any significant differences in these indicators regarding emergency settings. Our finding is not in line with the aforementioned findings [22]. Our finding may indicate that nurses in psychiatric settings are merely more likely to seek help for psychological disturbances because they can more easily recognize factors related to psychiatric wellbeing and have more positive attitudes toward mental health problems [48] than those working in medical and surgical settings. This might also indicate that psychiatric organizations and those providing emergency services have better tools to manage stressful work environments. The fact that psychiatric nurses are more likely to recognize these issues might also reflect on our finding of poor self-rated health among psychiatric staff, a finding that has emerged in previous studies, too [49]. Furthermore, certain types of violence such as bullying by staff members, which has been associated with employees’ wellbeing [14], might be more common in non-psychiatric settings compared to psychiatric settings when comparing occurrences found in separate studies (see, e.g., [50,51]). This situation might explain why nurses in medical and surgical settings suffer from psychological distress and sleep disturbances more often than nurses in psychiatric settings. However, the differences between the mean values of these wellbeing scores in psychiatric and non-psychiatric settings were small, as were the effect sizes. This raises a question about the relevance of our findings.

Contrary to our original assumption, we also report the novel finding that nurses working outside the psychiatric field are more likely to experience psychological distress and sleep disturbances in cases of patient aggression. Nurses working in psychiatric settings may be better educated on how to manage patient aggressive behavior [52,53] or they may have better coping mechanisms in these events. On the other hand, nurses working in psychiatric settings may be more hardened toward less severe forms of patient aggression, and therefore their psychological reactions are less severe than those of their counterparts. Our earlier studies have already shown that psychiatric nurses have reported in interviews that verbal assaults are not always recognized as violence [54], and patient aggression is rather unavoidable in their job [55,56]. On the other hand, a study conducted in Italy found that the association between experiences of verbal aggression and psychological problems were stronger among student nurses than among professional nurses [57], which might indicate that less experienced nurses have less resilience to workplace violence. When comparing occurrences in separate studies, nurses in non-psychiatric settings experience lower rates of, for example, patient-initiated verbal abuse compared to psychiatric nurses (see, e.g., [50,51]). It has been suggested that in non-psychiatric settings, perpetrators are mainly visitors, caregivers or relatives, whereas in psychiatric settings the perpetrators are mainly patients [58]. Therefore, non-psychiatric nurses might be less experienced than psychiatric nurses in managing this type of patient aggression and its consequences, which might explain the results that they are more likely to experience psychological distress and sleep disturbances in cases of patient aggression. However, this still raises the question of why nurses working in emergency settings are more likely to suffer from sleep disturbances in cases of physical assaults and armed threats. We may assume that, although certain types of aggression are more prevalent in emergency departments, education in the management of aggression and its consequences is lacking compared to that in psychiatric settings.

Our study raised two main questions, which remain unanswered. First, we need to ask whether poorer self-rated health and reduced perceived work ability among nurses working in psychiatric settings are signs of a serious hidden problem among staff in health services, which should urgently be considered. If nurses’ silent concerns cannot be identified, they may result in depression and medication use, something that has been found in our previous studies [22]. On the other hand, we need to ask whether nurses working outside psychiatric settings, who face aggressive events, are in more serious danger to suffer from poor psychiatric wellbeing and sleep disturbances. More research on this should be conducted. In any case, both problems identified in this study need to seriously be taken into account to ensure occupational safety and support the wellbeing of staff in their work.

This study has several limitations. First, the cross-sectional nature of the study does not allow us to make definite causal conclusions about the results. Longitudinal research itself with measurements at several time points is therefore needed in the future to verify our findings. Second, the study relies on self-reported questionnaires, which include the possibility of common method variance, and misunderstanding or modifying answers in order to give a more socially desirable response [59]. This is a case, especially in the retrospective evaluation of patient aggression during the 12 months prior to the measurement, which causes concerns due to recall bias or likelihood to underestimate the occurrence of aggression [60]. More objective data collection, such as organizations’ incident reports, could have been provided, although underreporting cannot be avoided in incident reports either [53,61]. On the other hand, all measures used in this study are widely used in large epidemiological studies, and their validity has previously been proven (see, e.g., [34,39,41]).

Third, the differences between the groups could have been affected by the large sample size, although the finding is not relevant in clinical practice. However, the sample size obtained in this study is representative, with a good response rate (72%) from various regions in Finland. This allows generalization of the results to Finnish healthcare services and abroad, keeping in mind the differences in the health systems.

## 5. Conclusions

Our results show that, compared to medical and surgical nurses, psychiatric nurses face patient aggression more often, but some types of aggression are more common in emergency settings. Subjective health and work ability levels among psychiatric nurses are worse than those among both of the non-psychiatric nursing groups, while the psychiatric wellbeing of psychiatric nurses is better, and they have less sleep problems compared to medical and surgical nurses. After exposure to patient aggression, psychiatric nurses have better psychiatric wellbeing and less sleep problems than non-psychiatric nurses. This suggest that more attention should be given in non-psychiatric settings for maintaining nurses’ wellbeing after exposure to patient aggression. Our study changes the previous understanding of which nursing fields are most taxing on nurses’ wellbeing. Our findings underline the importance of also evaluating and developing support (e.g., post-incident debriefing, clinical supervision and education) in non-psychiatric settings for maintaining nurses’ health and wellbeing after exposure to patient aggression, not only regarding physical aggression, but less severe forms of patient aggression, as well. Special attention should be given to emergency settings, where certain types of patient aggression are even more common than in psychiatric settings.

## Figures and Tables

**Table 1:** Demographic information of nurses working in different specialties (Finland, 2012).

	Psychiatry (N = 923)		Medical and Surgical (N = 4070)		Emergency (N = 295)	
	N	%	N	%	N	%
**Age (mean, SD)**	43.98	10.86	43.21	11.18	39.78	8.84
**Gender**	923		4070		295	
Male		25		5		14
Female		75		95		86
**Occupation**	923		4070		295	
Practical nurses ^a^		31		14		5
RN ^b^ SN ^c^		59		76		87
Head nurses		10		10		8
**The type of employment relationship**	923		4070		295	
Permanent		78		79		76
Temporary		22		21		24
**Years at current hospital (mean, SD)**	13.65	10.59	13.68	10.74	10.47	9.93
**Years in the current position (mean, SD)**	8.28	8.74	9.27	8.84	7.36	7.84
**Nature of the work**	923		4033		294	
Full-time work		95		90		94
Part-time work		5		10		6
**Form of regular working hours**	922		4045		295	
Regular daytime work		32		27		6
Two shifts ^d^		15		15		9
Three shifts ^e^		48		51		79
Night shift only		4		3		2
Other irregular work		1		4		4

^a^ Practical nurses = Mental health nurses, Mental nurses, Enrolled nurses, Practical nurses; ^b^ RN = Registered nurses; ^c^ SN = Specialized nurses; ^d^ Day and evening shift; ^e^ Day, evening and night shift.

**Table 2:** Comparison of nurses’ exposure to different types of patient aggression in psychiatric and non-psychiatric settings (Finland, 2012).

	Psychiatry (N = 923)		Medical and Surgical (N = 4070)			Emergency (N = 295)		
	N	%	N	%	*p* ^a^	N	%	*p* ^a^
**Experiences of at least one type of aggression**								
Yes	563	65	1374	36	**<0.001**	224	81	**<0.001**
No	297	35	2483	64		54	19	
**Assaults on ward property**								
Yes	440	49	514	13	**<0.001**	124	43	0.085
No	463	51	3492	87		165	57	
**Mental abuse**								
Yes	544	61	1141	29	**<0.001**	210	75	**<0.001**
No	343	39	2797	71		72	25	
**Physical assaults**								
Yes	333	38	820	21	**<0.001**	135	47	**0.005**
No	552	62	3143	79		153	53	
**Armed threats**								
Yes	41	5	36	1	**<0.001**	7	2	0.104
No	855	95	3957	99		283	98	

^a^ *p*-value, comparison with psychiatric nurses.

**Table 3:** Correlations of wellbeing scores (Finland, 2012).

	Self-Rated Health	Psychological Distress	Sleep Disturbances	Work Ability
Self-rated health	1	0.21	0.20	0.59
Psychological distress	0.21	1	0.30	0.26
Sleep disturbances	0.20	0.30	1	0.20
Work ability	0.59	0.26	0.20	1

**Table 4:** Comparison of nurses’ wellbeing in different settings (Finland, 2012).

	Psychiatry (N = 923)				Medical and Surgical (N = 4070)				Emergency (N = 295)			
	Mean	SD	N	%	Mean	SD	N	%	Mean	SD	N	%
**Self-rated health**	1.82	0.84			1.70	0.82			1.56	0.75		
Good			734	80			3375	84 ^a^			257	88 ^e^
Poor			185	20			674	16			36	12
**Psychological distress**	1.77	2.64			2.04	2.86			1.90	2.73		
No			750	81			3164	78 ^b^			233	79 ^f^
Yes			171	19			896	22			62	21
**Sleep disturbances**	3.30	1.49			3.48	1.45			3.15	1.41		
No			525	57			1993	49 ^c^			180	61 ^g^
Yes			398	43			2067	51			115	39
**Work ability**	8.15	1.52			8.35	1.48			8.78	1.25		
Good			693	75			3235	80 ^d^			254	87 ^h^
Reduced			228	25			822	20			39	13

^a^ *p* = 0.012, Cramer’s V 0.036; ^b^ *p* = 0.019, Cramer’s V 0.033; ^c^ *p* < 0.001, Cramer’s V 0.061; ^d^ *p* = 0.003, Cramer’s V 0.043; ^e^ *p* = 0.002, Cramer’s V 0.087; ^f^ *p* = 0.352, Cramer’s V 0.027; ^g^ *p* = 0.210, Cramer’s V 0.036; ^h^ *p* < 0.001, Cramer’s V 0.12.

**Table 5:** Odds ratios (OR) and 95% confidence intervals (CI) comparing wellbeing outcomes between nurses in psychiatric and medical and surgical specialties having encountered different types of aggression in their work (Finland, 2012).

Variable	Self-Rated Health				Psychological Distress				Sleep Disturbances				Work Ability			
	OR	95% CI	Wald (df)	*p* ^a^	OR	95% CI	Wald (df)	*p* ^a^	OR	95% CI	Wald (df)	*p* ^a^	OR	95% CI	Wald (df)	*p* ^a^
**At least one type of aggression**																
Psychiatric nurses vs.	1.01	0.68–1.50	0.004 (1)	0.950	0.55	0.37–0.81	9.13 (1)	0.003	0.65	0.48–0.89	7.33 (1)	0.007	1.01	0.70–1.47	0.005 (1)	0.946
Medical and surgical nurses	ref				ref				ref				ref			
**Assaults on ward property**																
Psychiatric nurses vs.	0.81	0.47–1.41	0.56 (1)	0.455	1.61	0.91–2.85	2.72 (1)	0.099	1.42	0.91–2.21	2.35 (1)	0.125	0.85	0.50–1.41	0.41 (1)	0.521
Medical and surgical nurses	ref				ref				ref				ref			
**Mental abuse**																
Psychiatric nurses vs.	1.13	0.68–1.87	0.22 (1)	0.638	0.39	0.23–0.66	12.17 (1)	<0.001	0.64	0.43–0.96	4.58 (1)	0.033	1.01	0.63–1.62	0.002 (1)	0.963
Medical and surgical nurses	ref				ref				ref				ref			
**Physical assaults**																
Psychiatric nurses vs.	0.78	0.47–1.30	0.94 (1)	0.777	0.87	0.52–1.46	0.27 (1)	0.601	0.69	0.46–1.04	3.12 (1)	0.078	0.88	0.55–1.41	0.29 (1)	0.590
Medical and surgical nurses	ref				ref				ref				ref			
**Armed threats**																
Psychiatric nurses vs.	1.92	0.56–6.59	1.06 (1)	0.302	1.04	0.36–3.01	0.006 (1)	0.937	1.03	0.39–2.71	0.003 (1)	0.959	1.60	0.56–4.55	0.78 (1)	0.378
Medical and surgical nurses	ref				ref				ref				ref			

^a^ Test of interaction.

**Table 6:** Odds ratios (OR) and 95% confidence intervals (CI) comparing wellbeing outcomes between nurses in psychiatric and emergency specialties having encountered different types of aggression in their work (Finland, 2012).

Variable	Self-Rated Health				Psychological Distress				Sleep Disturbances				Work Ability			
	OR	95% CI	Wald (df)	*p* ^a^	OR	95%CI	Wald (df)	*p* ^a^	OR	95% CI	Wald (df)	*p* ^a^	OR	95% CI	Wald (df)	*p* ^a^
**At least one type of aggression**																
Psychiatric nurses vs.	1.79	0.73–4.43	1.60	0.206	0.67	0.29–1.54	0.91	0.340	0.72	0.36–1.42	0.89 (1)	0.344	0.74	0.26–2.12	0.312 (1)	0.557
Emergency nurses	ref				ref				ref				ref			
**Assaults on ward property**																
Psychiatric nurses vs.	0.89	0.41–1.96	0.078 (1)	0.789	1.20	0.62–2.35	0.29 (1)	0.589	1.22	0.70–2.11	0.50 (1)	0.479	0.62	0.29–1.32	1.53 (1)	0.217
Emergency nurses	ref				ref				ref				ref			
**Mental abuse**																
Psychiatric nurses vs.	1.34	0.57–3.19	0.46 (1)	0.499	0.48	0.22–1.06	3.27 (1)	0.070	0.69	0.37–1.28	1.40 (1)	0.237	0.70	0.28–1.77	0.56 (1)	0.455
Emergency nurses	ref				ref				ref				ref			
**Physical assaults and armed threats ^b^**																
Psychiatric nurses vs.	1.22	0.55–2.72	0.24 (1)	0.624	0.99	0.51–1.95	0.00 (1)	0.987	0.57	0.33–0.98	4.06 (1)	0.044	1.06	0.50–2.25	0.03 (1)	0.873
Emergency nurses	ref				ref				ref				ref			

^a^ Test of interaction; ^b^ Physical assaults and armed threats are combined due to too few observations of armed threats.
